# Supplementary material for: Reconstruction of a regulated two-cell metabolic model to study biohydrogen production in a diazotrophic cyanobacterium Anabaena variabilis ATCC 29413
Source: PLoS One. 2020 Jan 24;15(1):e0227977. doi: 10.1371/journal.pone.0227977 (PMC6980584; doi:10.1371/journal.pone.0227977)
Supplement: S4 File — (DOC) [file pone.0227977.s004.doc]

**Reconstruction of a regulated two-cell metabolic model to study biohydrogen production in diazotrophic cyanobacterium *Anabaena variabilis* ATCC 29413**

Ali Malek Shahkouhi and Ehsan Motamedian

S4 File.

**Table A.** List of metabolic reactions active in vegetative cell or heterocyst.

| **Reference** | **Not present in** | **Pathway** | **Reaction equation** | **Reaction name** | **Reaction ID** |
| --- | --- | --- | --- | --- | --- |
|  | Het | Carbon fixation | o2[c] + rb15bp[c] -> 2 h[c] + 3pg[c] + 2pglyc[c] | ribulose-bisphosphate carboxylase | RBCh |
|  | Het | Carbon fixation | co2[c] + h2o[c] + rb15bp[c] <=> 2 h[c] + 2 3pg[c] | ribulose-bisphosphate carboxylase | RBPC |
|  | Het | Nitrogen metabolism | 2 h[c] + akg[c] + gln-L[c] + 2 fdxr-2:2[c] -> 2 glu-L[c] + 2 fdxo-2:2[c] | ferredoxin-dependent glutamate synthase | GLMS |
|  | Het | Carbon fixation | co2[c] + h2o[c] <=> h[c] + hco3[c] | HCO3 equilibration reaction | HCO3E |
|  | Het | Photosynthesis | 2 h[c] + pq[u] + h2o[u] + 2 photon[c] -> 2 h[u] + pqh2[u] + 0.5 o2[u] | photosystem II | PSII |
|  | Veg | Nitrogen metabolism | 16 atp[c] + 16 h2o[c] + n2[c] + 8 fdxr-2:2[c] -> 16 adp[c] + 16 pi[c] + h2[c] + 2 nh4[c] + 6 h[c] + 8 fdxo-2:2[c] | nitrogenase | NIT |

**Table B.** The predicted relative growth rates by two single-cell models in comparison with experimental values. According to the method presented in , mixotrophic growth rate for each carbon source was calculated and divided by autotrophic growth rate on bicarbonate.

| carbon source | Relative growth rate | | |
| --- | --- | --- | --- |
|  | Experimental | single-cell model (Malatinszky et al. ) | single-cell model (this study) |
| Putrescine | 0.7 | 1 | 1 |
| Bicarbonate | 1 | 1 | 1 |
| Urea | 1.86 | 1.22 | 1.38 |
| Glutamate | 4.5 | 4.6 | 8.36 |
| Glutamine | 10.3 | 4.6 | 8.36 |
| Acetate | 7.2 | 5.7 | 8.14 |
| Proline | 8.5 | 7.25 | 12 |
| Pyruvate | 7.36 | 7.7 | 7.66 |
| Maltose | 7.3 | 10 | 8.97 |
| Fructose | 7.8 | 10 | 9 |
| Sucrose | 9.93 | 10 | 9.1 |
| Glucose | 10.3 | 10 | 9 |
| Glycerol | 7.23 | 11.4 | 10.32 |

| (a) |
| --- |
| (b) |

**Fig A.** Distribution of a) 276 reactions in iAM957 that are not present in iJN678 and b) 158 reactions in iJN678 that are not present in iAM957 in various pathways.

**Fig B.** Effect of heterocyst percentage on growth rate using the two-cell models.

**Fig C.** Sensitivity of the error with respect to the C values using experimental growth data of Berberoglu et al. (Each average error and its variation represent the error distribution across all conditions and the error bars represent the standard error of the mean).

|  |  |
| --- | --- |
|  |  |
|  |  |

**Fig D**. Predicted growth rate versus expression level for some genes of photosystem II using the regulated two-cell model. The measured expression level for each gene is determined by a circle in all of the figures.

| (a) |
| --- |
| (b) |
| (c) |

**Fig E.** Maximum and minimum of intercellular exchange flux of sucrose and glutamate calculated using FVA for a) two-cell model of Malatinszky et al. , b) our two-cell model, and c) regulated two-cell model. Experimental data were obtained from and the labeled values indicate experimental irradiance (lux) and bicarbnate uptake rate (mmol/gDCW/h), respectively, for each data. The same colors were used for points with the same experimental data. For a fair comparison between sucrose and glutamate, the rates were converted to mmol carbon/gDCW/h and the predicted exchange rates of sucrose and glutamate were multiplied by 12 and 5, respectively.

**Fig F.** Double robusness analysis for heterotrophic growth of *Anabaena variabilis* on fructose with varying oxygen exchange and hydrogen production fluxes in vegetative cells.

**References**

[1] L. Curatti, E. Flores, G. Salerno, Sucrose is involved in the diazotrophic metabolism of the heterocyst-forming cyanobacterium Anabaena sp, FEBS Lett, 513 (2002).

[2] M.A. Kolman, C.N. Nishi, M. Perez-Cenci, G.L. Salerno, Sucrose in cyanobacteria: from a salt-response molecule to play a key role in nitrogen fixation, Life, 5 (2015) 102-126.

[3] A.N. Rai, P. Rowell, W.D. Stewart, Glutamate Synthase Activity of Heterocysts and Vegetative Cells of the Cyanobacterium Anabaena variabilis Kütz, Microbiology, 128 (1982) 2203-2205.

[4] J.-J. Park, S. Lechno-Yossef, C.P. Wolk, C. Vieille, Cell-specific gene expression in Anabaena variabilis grown phototrophically, mixotrophically, and heterotrophically, BMC genomics, 14 (2013) 759.

[5] M. Donze, J. Haveman, P. Schiereck, Absence of photosystem 2 in heterocysts of blue-green alga Anabaena, Biochim Biophys Acta, 256 (1972).

[6] D.G. Adams, Heterocyst formation in cyanobacteria, Current opinion in microbiology, 3 (2000) 618-624.

[7] D. Malatinszky, R. Steuer, P.R. Jones, A comprehensively curated genome-scale two-cell model for the heterocystous cyanobacterium Anabaena sp. PCC 7120, Plant physiology, 173 (2017) 509-523.

[8] H. Berberoğlu, N. Barra, L. Pilon, J. Jay, Growth, CO₂ consumption and H₂ production of Anabaena variabilis ATCC 29413-U under different irradiances and CO₂ concentrations, Journal of applied microbiology, (2008).

[9] R. Mahadevan, C. Schilling, The effects of alternate optimal solutions in constraint-based genome-scale metabolic models, Metabolic engineering, 5 (2003) 264-276.
