# Supplementary material for: Reconstruction of a regulated two-cell metabolic model to study biohydrogen production in a diazotrophic cyanobacterium Anabaena variabilis ATCC 29413
Source: PLoS One. 2020 Jan 24;15(1):e0227977. doi: 10.1371/journal.pone.0227977 (PMC6980584; doi:10.1371/journal.pone.0227977)
Supplement: Attachment — Submitted filename: Response to reviewers.docx [file pone.0227977.s007.docx]

Thank you for your valuable comments. We revised the paper according to your suggestions. Here are our answers to your specified questions.

6. Review Comments to the Author

Please use the space provided to explain your answers to the questions above. You may also include additional comments for the author, including concerns about dual publication, research ethics, or publication ethics. (Please upload your review as an attachment if it exceeds 20,000 characters)

Reviewer #1: Thanks for addressing all comments and suggestions, please make sure that you share the model as MAT, JSON, SBML, or XML.

The mat-files of the single-cell, two-cell, and regulated two-cell models for implementation in MATLAB are presented in supplementary files S3.

Reviewer #2: Satisfied with replies and changes in the manuscript

Reviewer #3: (No Response)

Reviewer #4: Reconstruction of a two-cell metabolic model to study biohydrogen production in a diazotrophic cyanobacterium Anabaena variabilis ATCC 29413

Overview:---------------

In the manuscript titled "Reconstruction of a two-cell metabolic model to study biohydrogen production in a diazotrophic cyanobacterium Anabaena variabilis ATCC 29413", the authors prepared a describe genome-scale network reconstruction (GENRE) of Anabaena variabilis ATCC29413 and applied it to the increase production of hydrogen. In general, authors have included many changes while some critical results I think still need to included in the manuscript. This includes comparison of metabolic networks of their model and the previously published photosynthetic bacteria (iJN678 and iSynCJ816: both available through BiGG)

More importantly, the authors need to provide additional simulations and benchmark their models with existing models of other cyanobacteria or other two-cell models. The only benchmarks, Figure 1 and 2, are still quite opaque and not very informative of the model as a parameter for theirsimulations. I have suggested possible changes that make the figure clear.

Overall, keeping the above points in mind, I think the authors need to undergo major revisions of their manuscript. Please find my detailed comments below.

Comments:---------
Major:

In the revision, Table 1 compares the size of the model in this study to previously published models. It is possible I didn't clarify what I was looking for. I think the best way for the reader to understand their model is for the authors to show differences in the models. This includes showing genomic metabolic differences between different organisms. This goes beyond comparing the size of the models which is not sufficient is not an indicator of differences. Which pathways are complete/incomplete in Anabaena but complete in other organisms/models. I feel these need to be discussed. One way authors can accomplish this (only a suggestion, authors can improvise) is by drawing a schematic of specific pathways and differently coloring the reactions which are differentially present in their models versus other models. This is an easy way to show that their model does indeed capture biological differences between organisms characterized in their respective genomes. Further, given that their ids are in BiGG database, this analysis/comparison can be easily performed.

There are many different reactions between the reconstructed metabolic models of cyanobacteria. For example, there are 340 reactions in our metabolic model that are not present in iJN678 and there are 220 reactions in iJN678 that are not present in our metabolic model. So, it is not possible to indicate the differences by drawing a schematic of specific pathways.

305-318: There are two points here:

a. The authors are talking about overprediction in high irradiance condition. Typically, models of photosynthetic organisms should behave as such when used with FBA: given the carbon uptake is constant, increase in light flux will increase growth upto a certain point, after that no amount of increase in light flux will cause any change in growth. Further, probably all of the existing models of photosynthetic organisms are likely to show this overprediction in high irradiance condition. After simulating the Two-cell model, I found that the model presented here captures both these aspects. The writeup in the manuscript represents the model in poor light. I would suggest that author find a different way of plotting Figure 2. One way to do this would be to adding two more subfigures in Figure 2: (1) Plot the light flux (x-axis) vs growth (y-axis) and find the ratio of uptake of light to HCO3; (2) Show what is this ratio in experimental conditions tested. Plotting this way does two things: (1) describes behavior of the model and (2) validates that behavior. Other models such as iJN678 and iSynCJ816 also do that. Overprediction in high irradiance condition means that organism reaches the plateau earlier than the model. This is expected as there likely gaps in the model; and hence, do not capture the entire range of bottlenecks. Discuss this point and show.

It should be mentioned that the bicarbonate uptake rate is not constant according to the experimental data [1] and it increases in high irradiances. Based on your comment we plotted the light flux (x-axis) vs growth (y-axis) at constant bicarbonate uptake rate of 1 mmol/gDCW/h (Figure R1). Then, we found that the minimum photon uptake rate for optimal growth is about 9 mmol/gDCW/h. However, the ratio of experimental bicarbonate to photon uptake rates (Figure R2) demonstrates that the minimum ratio is 23.3 in the experimental condition. So, the photon uptake rate is always more than the required rate predicted by FBA. So, unfortunately, this data does not answer to overprediction while the calculated shadow prices predicted the reason for overprediction.

If there were gaps in the metabolic network, the model should underpredict the growth. However, the model overpredicted and hence, there were intracellular constraints. We used the regulated two-cell model and shadow price to predict the bottleneck gene(s) under high irradiances.

Figure R1. Growth rate versus photon uptake rates predicted by the two-cell model (bicarbonate uptake rate is limited to 1 mmol/gDCW/h).

Figure R2. Photon uptake rate per bicarbonate uptake rate for 18 experiments carried out by [1].

b. In essence, authors are generating context-specific model for two-cell metabolic network. The authors are capturing only the photosystem II gene (PsbJ). However, with changes in light, many other intracellular metabolic genes are likely to get differentially expressed; and thus, are likely to come up as hits for whats happening in the model. There are many transcriptomics studies in photosynthetic bacteria suggesting this. I would suggest that author explain why there's only one hit. Also I think it is important to have a figure for how the expression of various genes from photosystem II change across different light conditions; thus, reinforcing their argument.

We used the easiest way and calculated shadow prices for all genes. Shadow price indicates the effect of one unit increase in gene expression on the predicted growth rate. Shadow price for other genes was zero and hence, the regulated two-cell model predicted that the measured expression level for other genes was adequate for growth. According to your comment, we plotted the growth rate versus expression level for some genes of photosystem II. It can be seen that only the expression level of PsbJ was not sufficient for optimal growth.

|  |  |
| --- | --- |
|  |  |
|  |  |

Figure 3. Predicted growth rate versus expression level for some genes of photosystem II using the regulated two-cell model. The measured expression level [2] for each gene is determined by a circle in all of the figures.

The explanations were added to the manuscript.

I understand authors used TRFBA because they developed it. But I think authors should discuss if a different context-specific model extraction algorithm could reproduce the same results.

TRFBA adds a constraint (Eq. 4) to the model for each gene with the measured expression level. So, we could use shadow price to indicate the effect of the expression level of each gene on growth and we could easily find the bottleneck gene. The predicted photoinhibition was in accordance with previous reports [3-6].

Our main aim in this research was the reconstruction and evaluation of a genome-scale metabolic model for *A. variabilis*. Evaluation of the integration algorithms is not our aim in this research. Some benchmarks are developed for evaluation of the integration algorithms (e. g. [7-9]). In our previous works [8, 10], we indicated that TRFBA is a successful algorithm for prediction of growth.

323-327: Please explain why the authors see this difference. What is the difference in the metabolic network of Malatinzsky et al and their model that yields transport of sucrose.

Interestingly, glutamate and sucrose intercellular exchange reactions are coupled in the two-cell model of Malatinzsky et al. [11] and the sucrose rate is maximized when glutamate is exchanged at the maximum rate. Furthermore, Figure S4 shows that maximum exchange rates for this two-cell model are always higher than those for our two-cell models and the rates unreasonably increase with an enhancement of irradiance. The maximal sucrose uptake rate of 46.8 mmol/gDCW/h is calculated at an irradiance of 16100 lux and bicarbonate uptake rate of 2.14 mmol/gDCW/h while this rate for our two-cell and regulated two-cell models is 1.29 and 1.12 mmol/gDCW/h, respectively. This indicates that there is an internal cycle of carbon in the two-cell model of Malatinzsky et al. [11]. This cycle circulates carbon sources between heterocyst and vegetative cells and the required energy for this circulation is provided by uptake of photon. Comparison between flux distributions for maximum and minimum intercellular exchange rates indicates that many reactions are involved in generating this internal cycle. Hence, Malatinzsky et al. [11] fixed the glutamine-glutamate exchange ratio to one that results in the reduction of the effect of this cycle.

The explanations were added to the manuscript.

330: What are "both" substrates: Glutamine-glutamate or sucrose-(?)

Glutamate and sucrose are correct. The manuscript was clarified.

331: Please explain this electron limitation and discuss the pathways the model uses to disspate excess electron at high irradiance.

We mean the limitation of PSII that previously explained. The manuscript was clarified.

As mentioned in the material and methods section, the maximum uptake rate of photon was set to the experimental values [1] because more irradiance does not necessarily increase the photon uptake rate. So, the model consumed photon with the required rate.

352: Of course, disabling PSII for a photosynthetic organism will not lead to generation of oxygen. This is not surprising and has been known for a long time now. The authors need to discuss differences in metabolic network of vegetative state versus heterocyst state. I recommend that authors provide this comparison for the reader.

This sentence is used for further emphasis. In the next sentences, we also explained more about the reason for the difference in the metabolic network of vegetative cells and heterocysts for oxygen. We also drew a schematic of the metabolic pathways and reactions in Figure 4 that shows the differences in the metabolic networks of heterocysts and vegetative cells.

370-373: The mechanism described appears to be similar to how photosynthetic organisms deal with light-dark (diel) conditions. Is it the same? Are there any differences? Please discuss. What if my map A. variabilis genes to Synechocystis and use iJN678 to simulate A. variabilis behavior in combination with TRFBA. Will I get the same result. Again for this reason, the authors need to compare the metabolic network of their model with other photosynthetic bacteria. Synechocystis model is most well studied, so I would use that.

Yes, it is the same and similar results for Synechocystis using its metabolic model (iJN678) are obtained without using TRFBA or mapping genes of *A. variabilis* to *Synechocystis*. This result is rational for every metabolic model of cyanobacteria because fructose provides both energy and carbon sources for the cell. However, in photoautotrophic conditions, bicarbonate is used as a carbon source and photon uptake that is necessary for energy generation produces oxygen.

The explanations were added to the manuscript.

Reference

1.	Berberoğlu H, Barra N, Pilon L, Jay J. Growth, CO₂ consumption and H₂ production of Anabaena variabilis ATCC 29413-U under different irradiances and CO₂ concentrations. Journal of applied microbiology. 2008.

2.	Park J-J, Lechno-Yossef S, Wolk CP, Vieille C. Cell-specific gene expression in Anabaena variabilis grown phototrophically, mixotrophically, and heterotrophically. BMC genomics. 2013;14(1):759.

3.	Yoon JH, Sim SJ, Kim M-S, Park TH. High cell density culture of Anabaena variabilis using repeated injections of carbon dioxide for the production of hydrogen. International journal of hydrogen energy. 2002;27(11-12):1265-70.

4.	Yoon JH, Shin J-H, Park TH. Characterization of factors influencing the growth of Anabaena variabilis in a bubble column reactor. Bioresource technology. 2008;99(5):1204-10.

5.	Agel G, Nultsch W, Rhiel E. Photoinhibition and its wavelength dependence in the cyanobacterium Anabaena variabilis. Archives of Microbiology. 1987;147(4):370-4.

6.	Markou G, Georgakakis D. Cultivation of filamentous cyanobacteria (blue-green algae) in agro-industrial wastes and wastewaters: a review. Applied Energy. 2011;88(10):3389-401.

7.	Machado D, Herrgård M. Systematic evaluation of methods for integration of transcriptomic data into constraint-based models of metabolism. PLoS computational biology. 2014;10(4):e1003580.

8.	Jamialahmadi O, Hashemi-Najafabadi S, Motamedian E, Romeo S, Bagheri F. A benchmark-driven approach to reconstruct metabolic networks for studying cancer metabolism. PLoS computational biology. 2019;15(4):e1006936.

9.	Pacheco MP, Pfau T, Sauter T. Benchmarking procedures for high-throughput context specific reconstruction algorithms. Frontiers in physiology. 2016;6:410.

10.	Motamedian E, Mohammadi M, Shojaosadati SA, Heydari M. TRFBA: an algorithm to integrate genome-scale metabolic and transcriptional regulatory networks with incorporation of expression data. Bioinformatics. 2016;33(7):1057-63.

11.	Malatinszky D, Steuer R, Jones PR. A comprehensively curated genome-scale two-cell model for the heterocystous cyanobacterium Anabaena sp. PCC 7120. Plant physiology. 2017;173(1):509-23.
