# Supplementary material for: Reconstruction of a regulated two-cell metabolic model to study biohydrogen production in a diazotrophic cyanobacterium Anabaena variabilis ATCC 29413
Source: PLoS One. 2020 Jan 24;15(1):e0227977. doi: 10.1371/journal.pone.0227977 (PMC6980584; doi:10.1371/journal.pone.0227977)
Supplement: Attachment — Submitted filename: Response to reviewers.docx [file pone.0227977.s008.docx]

Thank you for your valuable comments. We revised the paper according to your suggestions. Here are our answers to your specified questions.

Review Comments to the Author

Please use the space provided to explain your answers to the questions above. You may also include additional comments for the author, including concerns about dual publication, research ethics, or publication ethics. (Please upload your review as an attachment if it exceeds 20,000 characters)

Reviewer #2: No additional comments

Reviewer #4: Reconstruction of a two-cell metabolic model to study biohydrogen production in a diazotrophic cyanobacterium Anabaena variabilis ATCC 29413

Overview:---------------

The authors have for the most part addressed all of my previous concerns. But I think the one analysis that I reiterate here are important addition to this paper and contains important information about the meabolic model reconstructed here. Keeping this in mind, I recommend minor revisions.

Comments:---------
Major:

Clearly, the authors agree that there are difference between their model and iJN678. As I had stated in the previous round that pathway specific differences need to be discssed between their model and existing model(s). Since, this is the first time this model is being published, it comes on the authors to elaborate on this. The authors only need pathway annotations from the models to prepare a bar graph where each bar represents one pathway. This is clearly possible and relatively quickly.

According to your comment, we carefully matched the reactions of the two models and eliminated the same reactions under different names. Two bar charts (Figures S1 (a) and (b), Supplementary file S4) were plotted to indicate the distribution of different reactions in the metabolic pathways. Furthermore, more explanations were added to the manuscript.
